# A Systematic Approach in Developing Management Workforce Readiness for Digital Health Transformation in Healthcare

**DOI:** 10.3390/ijerph192113843

**Published:** 2022-10-25

**Authors:** Mark Brommeyer, Zhanming Liang

**Affiliations:** 1 College of Business, Government and Law, Flinders University, Adelaide 5042, Australia; 2 College of Public Health, Medical and Veterinary Sciences, James Cook University, Townsville 4811, Australia

**Keywords:** health service managers, competency frameworks, capacity building, digital health, health informatics, health workforce, health management degrees

## Abstract

Background: The COVID-19 pandemic has sped up digital health transformation across the health sectors to enable innovative health service delivery. Such transformation relies on competent managers with the capacity to lead and manage. However, the health system has not adopted a holistic approach in addressing the health management workforce development needs, with many hurdles to overcome. The objectives of this paper are to present the findings of a three-step approach in understanding the current hurdles in developing a health management workforce that can enable and maximize the benefits of digital health transformation, and to explore ways of overcoming such hurdles. Methods: A three-step, systematic approach was undertaken, including an Australian digital health policy documentary analysis, an Australian health service management postgraduate program analysis, and a scoping review of international literatures. Results: The main findings of the three-step approach confirmed the strategies required in developing a digitally enabled health management workforce and efforts in enabling managers in leading and managing in the digital health space. Conclusions: With the ever-changing landscape of digital health, leading and managing in times of system transformation requires a holistic approach to develop the necessary health management workforce capabilities and system-wide capacity. The proposed framework, for overall health management workforce development in the digital health era, suggests that national collaboration is necessary to articulate a more coordinated, consistent, and coherent set of policy guidelines and the system, policy, educational, and professional organizational enablers that drive a digital health focused approach across all the healthcare sectors, in a coordinated and contextual manner.

## 1. Introduction

In the rapidly changing, digitally-connected healthcare environment, health service managers need capabilities and relevant competencies to enable data-driven, strategic and operational decision-making [1,2,3], and the capacity to lead and manage digital health transformation. Health service managers must tackle the challenges of unprecedented growth in digital health literacy within this period of systemic transformation, and be proficient in planning and managing the digital tools and technologies across this shifting landscape [4,5].

### 1.1. COVID-19 and Digital Health Transformation in Healthcare

The COVID-19 pandemic has pressured the adoption of innovation in service delivery within healthcare systems and organizations globally, including the rapid adoption of digital health technologies, as healthcare practitioners and systems needed to adapt to new ways of working, with omnipresent social distancing and travel restriction requirements the norm. As witnessed in Europe and the United Kingdom, “many countries have adopted digital-first strategies, remote monitoring and telehealth platforms to enable healthcare provision without physical interactions” [6] (p. 1). In addition, digital health systems have also played a critical role in support of public health policies [7,8] and improving communication and information in healthcare; COVID monitoring and surveillance; health services provision, and vaccination bookings, recording, and monitoring [9]. In the United States of America, elements that supported the rapid adoption of digital health solutions and innovation during the pandemic included “technology innovations and policy prescriptions” (p. 9), including “right-sizing of regulation” (p. 8), for example, recalibrating virtual medical visit requirements under the Health Insurance Portability and Accountability Act [10]. In Australia, the Federal government’s pandemic response included implementing the required policy and funding arrangements for digital health innovation to be used across the country [11].

Globally, in August 2020, the G20 Riyadh Declaration on Digital Health was formulated, which presented nine recommendations on digital health to address the challenges of the COVID-19 and future pandemics. This included a “consensus on high-priority issues identified within 5 themes: team, transparency and trust, technology, techquity (the strategic development and deployment of technology in health care and health to achieve health equity), and transformation” [12] (p. 1). The fast growing cross-sector, digital health transformations highlight the pressing need to develop a workforce equipped with the knowledge, skills, and capabilities in deploying and managing digital technologies vital to meet the current and future public health challenges, in a timely and systematic manner [12].

### 1.2. Evidence on Workforce Development Needs

Success in healthcare innovation and transformation necessitates a health workforce with the required understanding and new skill sets, which does not happen overnight and is a continuous improvement process. Using the introduction of electronic health records (EHR) as an example, after being broadly implemented in the healthcare system, in particular the hospital sector for more than a decade, mounting evidence indicates that EHRs have not been adequately utilized by clinicians to guide clinical decision-making [13]. Clinicians’ lack of understanding of the benefits of EHRs, their frequent encounter with difficulties in access, and the perceived lack of effectiveness and efficiency of EHR usage, were the three major reasons for the lack of EHR take-up [14,15].

Empirical evidence further identified that leaders’ lack of awareness of their role in mobilizing and supporting staff and collaborating between key stakeholders in implementation, and inadequate understanding of the benefits of EHR, were two of the barriers to EHR success [1,16,17]. Not having the understanding of how EHRs can benefit and guide practices, and not having the technical skills required to work in the EHR context and utilize the digital data to guide decision-making, were the two key areas requiring targeted training and development prior to and during the introduction and implementation process [13].

### 1.3. Policy Guiding Digital Health Workforce Development

Overall health workforce development should be fundamentally driven and supported by workforce policy with allocated funding and resources [18], and concomitant efforts at system, institution, organization, and individual levels. In Australia, the Australian Digital Health Agency (ADHA) provides national policy direction and targeted funding for digital health, including the development of the National digital health workforce and education roadmap [19]. The roadmap clearly specifies the need to acquire a variety of digital literacy and baseline capabilities across the healthcare workforce, and suggests that the digital knowledge and skills required, will differ based on the diverse digital health roles and service delivery requirements throughout the healthcare system. They have also identified eight digital profiles, recognizing some consistency of digital capabilities required across health workforce roles, contexts or environments.

Two of the profiles: ‘leadership and executive profile’, and ‘the business, administration, and clinical support digital profile’, are both of particular importance as capable leaders and managers of a digitally-enabled workforce are key factors in successfully adopting and managing digital health transformation.

Unlike other health professions, health service management is not regulated, resulting in no specific requirements for management qualifications. Management competency improvements are often not embedded in regular management performance appraisals. This results in inadequate incentives for continuous, informal management training and development, which are both costly and time-consuming. Hence, in order to develop overall management workforce competence, political will and policy direction are required. International studies [20,21,22,23,24,25] have also highlighted that policy and system-level factors are crucial for healthcare management workforce development, in ensuring digital health adoption success. These factors include ensuring that a comprehensive digital health policy clearly aligns with the organization’s strategic goals, that support and investment in socio-economic and regulatory impact assessments of digital technologies are provided, and the privacy and integrity of digital data are assured. Clear governance rules and regulations regarding the use of digital technologies, supported by contextually applied technology implementation and outcome measurement training, are also critical.

### 1.4. The Role of Universities, Professional Institutions, and Organizations in Workforce Development

In addition to policy direction and incentives, the provision of skill development for the health workforce relies on the combined efforts between university programs, professional institutions, and individual healthcare organizations. Using the health service management (HSM) workforce in Australia as an example, at the institutional level, its development relies on 21 university programs such as the Master of Health Administration (MHA) and Master of Health Service Management (MHSM) awards, and professional institutions: the Australasian College of Health Service Management (https://www.achsm.org.au: accessed on 1 September 2022) and Royal Australasian College of Medical Administrators (https://racma.edu.au: accessed on 1 September 2022). Other member-based professional institutions, such as the Australian College of Nursing (https://www.acn.edu.au: accessed on 1 September 2022), Australian College of Rural and Remote Medicine (https://www.acrrm.org.au: accessed on 1 September 2022), and Australasian Institute of Digital Health (https://digitalhealth.org.au: accessed on 1 September 2022), also provide management development opportunities to specific professions.

In Australia now, there are a slowly developing number of digital health postgraduate program offerings, but they are not specifically targeting health service managers, and the capacity in developing HSM is limited [26]. Digital health transformation requires competent managers with the capacity to lead and manage, with relevant competencies that enable data-driven, strategic, and operational decision-making [1,2,3]. Health service managers must tackle the challenges of unprecedented growth in digital health literacy, within this period of systemic transformation, and be proficient in planning and managing the digital tools and technologies across this shifting landscape [4,5]. The COVID-19 pandemic has accelerated digital health adoption in healthcare for safer and more efficient service delivery in a timely manner. Such fast transformation does not allow much room for ‘learning on the job’ for health service managers, therefore, a holistic approach incorporating different upskilling mechanisms in addressing the health management workforce development needs has to be reconsidered.

### 1.5. Aims and Objectives

Health management workforce development needs are context-sensitive, and heavily influenced by the existing political will and policy direction. Hence, in order to develop a management workforce with the capacity to lead and manage digital health transformation in Australia, the current efforts at system and institution levels need to be identified, and learning from international experience and their applicability in the Australian context, are required. In addition, a systematic and universal guiding framework for overall management workforce development needs to be developed, clearly specifying how efforts at various levels interact.

The purposes of this paper are to examine the current approaches in, and hurdles to, developing the Australian HSM workforce in the context of digital health transformation, and to identify strategies that can develop a digitally enabled health management workforce in the digital health era. This will lead to the development of a guiding framework for short- and longer-term health management workforce development and transformation requirements.

## 2. Materials and Methods

A three-step, qualitative approach has been adopted which includes: (1) documentary analysis of the Australian digital health policy; (2) analysis of the Australian HSM postgraduate programs and mapping the programs against the digital health-related competencies, and (3) a scoping review of international literatures focusing on strategies to develop HSM workforce capacity in the digital health context. This was guided by the evidence-informed approach to the development of workforce competency frameworks in healthcare professions, as described by Batt et al. [27] (p. 914), who indicate that “While there is no guidance on what specific methods to use, when to use them, or how to use them, there is consensus that in order to increase the validity and utility of competency frameworks, a combination of approaches may be necessary, akin to a process of triangulation”. The study analyzed the Guide to Developing Competency Standards for Professions by Heywood et al. [28], describing the first of five stages, which included examining: (1) the existing information from government reports, (2) studies undertaken by the profession, and (3) curriculum documents. This provided the basis for guiding the adoption of the three-step approach used for this study: (1) an analysis of Australian health policy documents, (2) a scoping review of international literature, and (3) an analysis of Australia’s health services management postgraduate programs.

### 2.1. Digital Health Policy

Digital health and workforce policy drivers were analyzed from twelve national organizations that are pertinent to digital health and workforce development in Australia (listed in Appendix A). These policies were identified by national expert digital health working groups, led by the Australian Digital Health Agency, through undertaking environmental digital health policy, capability, and competency framework scans. As Cardno explains, “As a qualitative research method, documentary analysis is often chosen as a second or supplementary way of collecting data in order to add rigour to a study through a multi-method form of triangulation” [29] (p. 626).

The twelve identified digital health government, educational, and workforce registration credentialing policy frameworks, were analyzed for digital health capability statements and keywords, competency domains, and professional certification requirements. These were then validated using a competency and narrative analysis review by the two researchers with domain expertise, for congruence.

### 2.2. Postgraduate Healthcare Management Programs

This research builds on a previous study, where the Australian Health Informatics Competency Framework’s 50 health informatics competency statements were mapped to the 21 postgraduate health management programs offered domestically in Australia, that received accreditation from the Australasian College of Health Service Management (ACHSM) by course purposes and learning outcomes of core subjects [30]. This followed the ‘Steps Used to Effectively Map Preexisting Courses to Competency Sets’ approach, developed by the University of Washington School of Public Health’s Northwest Center for Public Health Practice (NWCPHP), as this has demonstrated a high level of confidence in the accuracy of the process for mapping competencies to its courses [31].

Both authors then independently analyzed the current 17 master’s degree programs (listed in Appendix B), adopting a modified ‘Steps Used to Effectively Map Preexisting Courses to Competency Sets’ approach, developed by the University of Washington School of Public Health’s Northwest Center for Public Health Practice (NWCPHP) [31].

### 2.3. Scoping Review

A scoping review of the literatures was conducted between 2020 and 2022. The initial focus was to identify the current efforts in developing a digitally enabled HSM workforce. Considering the small number of papers identified, the search of literatures was later expanded to cover all efforts in developing the health management workforce with key capabilities for the demonstration of required management competencies. The review was guided by the five-step framework defined by Arksey and O’Malley [32] including the following steps: (1) defining a research question, (2) identifying relevant studies, (3) selecting and confirming empirical studies, (4) data extraction, and (5) collating, summarizing, and reporting results.

The review searched the following databases: Scopus, ProQuest, Web of Science, ACM Digital Library, CINAHL, PubMed, Google Scholar, and ProQuest Dissertations. The scoping review used the following keywords: ‘health informatics’, ‘digital health’, ‘electronic health’, ‘competencies’, ‘capability’, ‘proficiency’, ‘qualification’, ‘certification’, ‘health manager’, ‘health executive’, ‘health administrator’, ‘training’, ‘education’, and ‘professional development’, which were confirmed in consultation with an academic research librarian at James Cook University. A PRISMA approach [33] was used for eligibility screening. The review searched for empirical articles published in the English language since the year 2000, that provided information addressing the objectives as detailed above.

The key findings of the review were extracted from the eligible papers, which were subject to content analysis in order to identify the essential themes relevant to the search focus.

## 3. Results

### 3.1. Policy Analysis

The analysis of digital health and workforce policy drivers from the above mentioned twelve national organizations found that for the digital health capabilities required for a competent, nationally certified, and registered healthcare workforce, there are disparate, differentiated, and diverging requirements included in these national policy frameworks, which guide the development of digital health capabilities across the healthcare workforce. The core digital health capabilities, foundational to all the healthcare workforce, could focus on domains such as Digital Professionalism, Leadership and Advocacy, Data and Information Quality, and Information Enabled Care, and Technology [34]. The contextualized roles, e.g., HSM, require discipline-specific competencies to be demonstrated for increased proficiency across healthcare settings.

### 3.2. Postgraduate Healthcare Management Program Analysis

Ten out of the 17 postgraduate programs offered digital health subjects, either as a major specialization or as elective topics. These subjects commonly address the following competency areas, as included in the Australian Health Informatics Competency Framework:

(1) digital literacy,
(2) use of information technologies in the health context,
(3) awareness of new and emerging technologies in healthcare,
(4) technology-enabled and data-driven operational and strategic decision-making,
(5) future and current applications for digital health including the role of government, trends in big data, virtual, and telehealth,
(6) use of technology for sustainable healthcare,
(7) digital innovation and data analytics, and
(8) digital transformation of healthcare delivery.

These programs cover a range of operational and technical, program, project and change management capabilities for implementing digital tools and technology. However, the specific competencies required for leading and managing the workforce through digital transformation need to be included. This may include system, organizational, and team management skills, aligning the digital tools and technologies in support of required business and clinical, evidence-informed decision-making.

In Australia, there is now a Master of Digital Health program: (https://www.latrobe.edu.au/courses/master-of-digital-health: accessed on 2 September 2022), along with eleven Graduate Certificates in Health Informatics and Digital Health offerings. The master’s degree focuses on evidence-based practice in digital health, implementing and evaluating contemporary digital health solutions, digital health safety and patient outcomes, with a primary focus on digital health consultants, managers and researchers. Whereas, the graduate certificates have a varied and diverse range of subjects targeting digital health skills development for the clinical, operational, and technical workforce, at a discipline-specific and foundation level.

### 3.3. Scoping Review

**Figure 1:**
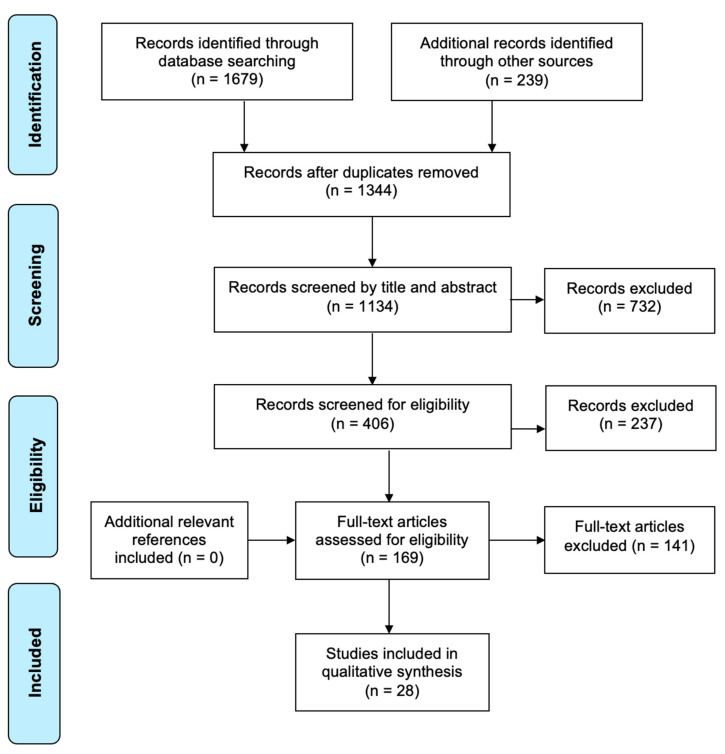
PRIMA flow diagram.

An initial search conducted in 2020 and 2022 generated 1679 publications, and after duplicates were removed, 1344 plus 239 publications were included for title screening, leading to 406 articles included for abstract screening by two reviewers. In total, 169 papers were deemed relevant for full-text review, leading to the inclusion of 28 papers that discussed strategies for developing the HSM workforce, which were then included in the data extraction and qualitative content analysis. The overall outcome of the review process is detailed in Figure 1 below, guided by the framework outlined by Arksey and O’Malley [32].

Twenty-four out of the 28 papers were published after 2010 including four published between 2020 and 2022. These papers presented results of the studies conducted in multiple countries located in Europe, Southern Asia, Northern America, and the Western Pacific with about 30% of them conducted in the USA and 20% of them in Australia. All of these papers presented some evidence on ways of developing health service manager’s competence and management capacity. Twelve papers presented results of the evaluation of various leadership and management training to different professional groups, including clinicians, nursing staff, and different types of management positions. These evaluation studies presented positive outcomes in improving managers’ management competencies, and the analysis confirmed that training programs targeting specific competency areas could develop managers’ competency and management capacity, and institute positive change [35,36,37,38,39,40,41,42,43,44,45,46]. Leadership and management training has been proven a key ingredient in health system strengthening [40]. In addition to the importance of training and development, five other strategies in developing the management workforce’s competency and capacity have also been discussed and confirmed in the studies, which are detailed in Table 1.

In addition to developing health management workforce competency and capacity, efforts in enabling health managers to lead and manage in the digital health context have also been discussed and confirmed in the literature, and presented as seven key factors that enable health management workforce development, as detailed in Table 2.

## 4. Discussion

A digitally enabled management workforce is crucial for health service organizational and care delivery success [13]. The three-step approach undertaken in this study has confirmed some recent efforts in meeting such workforce development needs. However, a systematic and universal guiding framework for overall management workforce development clearly specifying how efforts in system, institution, organization and individual levels interact, has yet to be identified. The findings of the three-step approach confirmed the pressing need to incorporate digital health-related competencies in the existing training curriculum for health services managers, further, it has highlighted the important role of short-term targeted training in developing a health management workforce that is digital health ready. The policy settings for the digital health management workforce also need to provide an increased focus on leading and managing digital transformation, and the competencies that can inform organizational capability, professional credentialing, postgraduate curricula, and industry certifications [26]. Factors that enable the development of the requisite health management workforce capabilities and system-wide capacity may include appropriate policy, supportive organizational systems and structure, aligned education and training offerings, and the capacity of the organization to support digital health adoption [40,45,56,57].

There has been an increasing movement to develop management competency frameworks, against which health service managers can apply for credentialing and certification. These frameworks are evolving, recognizing the fast-moving environment in which healthcare is delivered. It was also evidenced that there has been a paucity of digital health competencies embedded within these HSM competency frameworks. This study also highlighted the requirement to both develop a competency model to guide the required digital health competencies for health service managers [47,53], as well as embed competency assessment into management competency development processes [46,47,48]. The need to include management and leadership competencies that focus on enabling system-wide transformation in the current digital context, was also evidenced [26].

**Figure 2:**
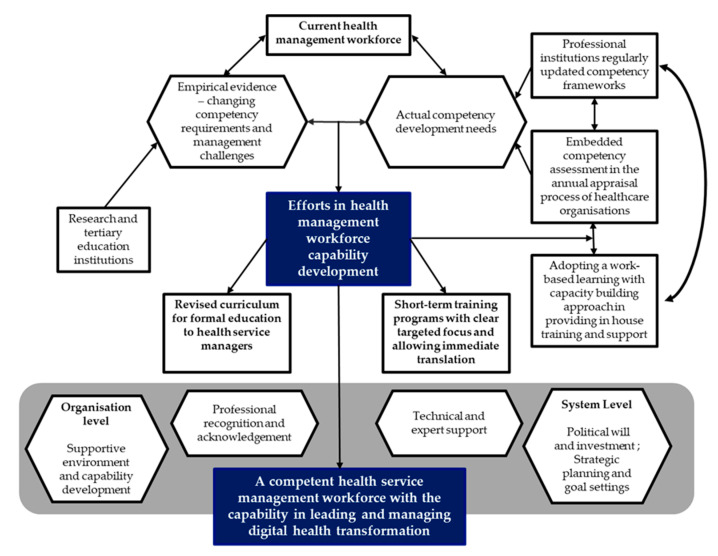
Framework for developing the HSM workforce in the digital health context.

This paper used a three-step approach, supported by empirical research, in guiding the creation of a conceptual framework (see Figure 2) for developing the health service management workforce capacity, in context. A contemporary approach to using new knowledge is presented, in developing this conceptual framework. Information represented, as a result of the scoping review, provides an evidence-based process to confirm the relevance and importance of existing knowledge, to guide the policy and practice implications, frameworks, directions, and workforce recommendations.

### 4.1. Efforts in Developing a Digitized HSM Workforce

Efforts in developing a digitized HSM workforce are evident at multiple levels. Digital health and workforce policies have been developed [18]; professional institutions have been fast to recognize the additional skill development requirements by adding new competencies into the existing training frameworks. New postgraduate degrees focused on the systematic development of digital health professionals have also been developed and offered by a small number of Australian universities. However, whether the policies and revised frameworks have been translated into guiding the development of the HSM workforce that is digital health ready, remains unclear [26].

Although formal education is important in its ability to systematically develop one’s overall professional competence, the immediate upskilling of the HSM workforce relies on short-term professional development programs that allow immediate translation into practice [36,37,38,39,40,52]. This is particularly true when evidence indicates that specific competencies relevant to leading and managing digital health transformation are required to be developed among health service managers [62]. Short-term training targeting identified gaps in competencies is more appealing and relevant to health service managers for several reasons: workload, time availability, and level of required commitment.

Literature has confirmed that management and management competency is context- sensitive and influenced by the different nature of management positions and management levels [47,48]. A number of papers discussing the evaluation results of training programs reinforced the importance of taking organizational culture into consideration when designing training programs [45,55], hence, a work-based and action-learning approach was suggested [38,52]. This is certainly much easier to be adopted through short-term training programs rather than formal education, which was subject to strict university rules and regulations.

The higher the management levels, the higher the proportion of managers who would have acquired postgraduate qualifications [47,48], hence, short-term programs, without fulfilling other degree requirements, may be more attractive to senior management levels. On the other hand, entry- and middle-level managers may take on postgraduate study to increase competitiveness in advancing their management careers, hence, ensuring that the existing postgraduate curriculum addressing the competency development needs of their targeted student cohorts, must become one of the annual quality assurance processes for all postgraduate programs. In the case of digital health readiness, incorporating competencies that are necessary for managers to lead and manage in the digital health era, within the existing educational framework, is a very important step to take [35]. Professional institutions, such as ACHSM in Australia, have the responsibility to support and ensure the accredited formal education programs for health service managers, and are responsive to the development needs of the changing workforce [63].

### 4.2. The Importance of Strategic Planning, Support, and Removing Obstacles

It is important to develop health service managers’ digital health competencies, but this is only part of the answer to developing a workforce capable of leading and managing digital health transformation. Leading and managing digital health transformation is an emerging and essential requirement for health service managers, in addition to their existing core responsibilities. No training can immediately fully develop their competencies in strategically utilizing the ever-changing digital health tools and technologies, applying data governance [64], developing the right systems for data management [65], and having an organization-wide awareness of required digital tools and technologies [66]. Furthermore, a sound understanding of how digital health systems promote quality care [67], as well as personal health information privacy and security principles, are key attributes required of successful health service managers [5,65]. Technical expertise and organizational support are also necessary.

It is equally important to develop the health workforce’s overall understanding of digital health and how it can be used in context. This can be achieved through integrating digital health capabilities in all workforce activities, including systematic planning and embedding of professional development needs in long-term individual and organizational digital health goals [51]. The focus on developing foundational levels of digital literacy across the health workforce, and the depth of the requisite knowledge, needs to be based on the different digital health roles and people within the system [19].

System-level guidance in what competencies should be covered by formal education and professional institutions is also required. Digital health skill development amongst health service managers should be a coordinated effort, rather than relying on individual programs or organizations to fill the gaps, based on the expertise that they have.

### 4.3. A Holistic Approach toward HSM Workforce Development to Enable Digital Health Transformation

As discussed above, short-term training targeting the improvement of specific competencies, is one key strategy for the development of a competent and capable health management workforce. However, current training for managers is mostly designed and offered on an ad hoc basis and is based on a ‘what I believe is important’ mentality, by those who offer the training. A systematic approach to integrating the specific competencies required for leading and managing the workforce through digital transformation needs to be included in formal education, continuing professional development, and professional association recognition and certifications. This should include developing the system, organizational, and team management skills, as well as aligning the digital tools and technologies to support the necessary business and clinical, evidence-informed decision-making [61].

Competency assessment can identify an individual’s competency gaps and training needs via various processes such as self-assessment and 360-degree assessment [48,53]. Empirical evidence has also suggested that self-assessment is a very beneficial self-educational process leading to actual knowledge and skills improvement, and also an important motivating factor for self-learning [68,69]. Considering all key strategies and factors as discussed above, this paper proposes the following framework (Figure 2) to guide overall health management workforce development in the digital health era.

The framework suggests a national collaboration to articulate a more coordinated, consistent, and coherent set of policy guidelines that foster digital health and workforce development. Any national, digital health policy guidance and directions should be underpinned by relevant and contextualized global policies, for example, the World Health Organization guideline on digital interventions for health system strengthening [70].

Ongoing and collective efforts are required in developing a national, core set of digital health competencies for the healthcare management workforce that guide a more consistent curriculum and set of course offerings, which could then be accredited via a nationally endorsed, digital health capability framework, to better guide postgraduate workforce development and relevant professional development offerings. Recognizing that in Australia, as in many countries around the world, significant work has been undertaken, and is ongoing, to produce and ratify national digital health capability frameworks. These could also include reference to relevant and contextualized global frameworks, for example, the World Health Organization’s guidance on digital education for building health workforce capacity [71].

In the rapidly changing healthcare environment, the professional development needs of the HSM workforce cannot be met without specific efforts in understanding the changing requirements. The scoping review only identified six papers most relevant to HSM development in the digital health context [50,51,57,59,60,61]; more research is needed to generate up-to-date evidence to guide developing a competent HSM workforce, and to address the challenges facing health service managers with the capacity to lead and manage in the digital health era.

## 5. Conclusions

Sustainable, quality, and safe healthcare services require a management workforce equipped with contemporary leadership and management capabilities. With the ever-changing landscape of digital health, health service managers are required to lead and manage in times of system transformation. Digital competencies are required for the HSM profession as well as the general healthcare workforce, which needs collaborative efforts across healthcare organizations, government, educational, and professional institutions.

This paper not only confirmed the urgent need to incorporate digital health-related competencies in the existing training curriculum for health service managers, but also highlighted the important role of short-term, targeted training in developing a health management workforce that is digital health ready, and the efforts that are required to enable managers to lead and manage in the digital health space. The proposed framework, for overall health management workforce development in the digital health era, suggests that national collaboration is necessary to articulate a more coordinated, consistent, and coherent set of policy guidelines that foster digital health and workforce development.

Management workforce capacity-building needs to adopt a holistic approach to developing the requisite HSM capabilities and system-wide capacity, which may include appropriate policy, supportive organizational systems and structure, and aligned education and training offerings. HSM workforce development is not a one-off effort. It requires system-level investment, support, and recognition, and collective efforts in removing the barriers and hurdles to the ongoing development of required digital health competencies and capabilities.

## Figures and Tables

**Table 1:** Five key strategies for developing health management workforce competency and capacity.

Strategy	Details
Competency assessment [46,47,48]	Embedding competency assessment into management competency development processes.
Competency models [44,46]	Developing a competency model to guide in developing competent HSM.
Formal development [49,50,51]	Providing formal and comprehensive HSM development opportunities to managers with three considerations. *
Short-term training [36,37,38,39,40,52]	Providing short-term training programs targeting specific competency areas with seven considerations as management competency is context- sensitive. **
Work-based development [38,52,53,54,55]	Adopting a work-based learning and capacity-building approach in providing training and support within the organization.

* Analysis of the literature identified three key considerations for providing formal and comprehensive HSM development opportunities to managers: (1) Incorporating digital health competencies into the HSM teaching curriculum; (2) Integrating required digital health curriculum content with theory and practice to allow immediate translation; (3) Allowing knowledge to be articulated to the requirements at organizational, sectorial, and institutional levels. ** Short-term training programs targeting specific competency areas should have the following considerations: (1) Meeting the needs of different management levels within the organization; (2) Taking the size of the hospitals into consideration, as additional support may be required for small hospitals; (3) Leadership and management training and development needs to reflect local culture, hence work-based and action learning approaches should be adopted; (4) Recognizing management competency development is an iterative, dynamic, and complex process; (5) Keeping capacity-building approach in mind when developing training programs; (6) Recognizing complex leadership challenges can be a source of significant experiential learning for individuals and groups, hence, articulating and reflecting on experiential learning can elucidate the skill, knowledge, and judgments embedded in management practice; (7) A progressive and staged learning process contributes to skill consolidation.

**Table 2:** Seven key factors that enable health management workforce development.

Factors	Details
Acknowledement and recognition [39,45]	Acknowledging health service managers’ new responsibilities and the efforts of HSM in acquiring new skills. Provide formal recognition of the required competencies via certification or provision of credentialing.
Adopting innovation [45]	Organization’s capacity in adopting innovation and support that assists HSM in adopting innovation.
Supportive environment [56]	A supportive environment in innovation and efforts in addressing system and individual level constraints, allowing managers to facilitate the adoption of health innovations and learn from the process.
System level support [40,57]	Provide high level support and political will in developing leadership and management across sectors and organizations.
Specialized expertise [58]	Support from experts with required digital health and health informatics expertise is provided with complementary information to explain difficult digital health concepts and understand digitized data for decision-making.
HSM workforce investment [51,59,60,61]	Investment in developing the health informatics and digital health workforce is critical. Managers cannot lead a workforce that are not yet ready.
Systematic integration [51]	Invest in systematic planning and development of professional practice in the health professions and integrate the professional development need in long-term ehealth and clinical informatics goals.

## Data Availability

Not applicable.

## Appendix A. Twelve Analyzed Digital Health Policies

(1) Australasian College of Health Service Management (2022). Master health service management competency framework, 2.0.
(2) Australian College of Rural and Remote Medicine (2021). ACRRM Fellowship Training Program.
(3) Australian Digital Health Agency (2020). National digital health workforce and education roadmap.
(4) Australian Digital Health Agency (2020). Nursing and Midwifery Digital Health Capability Framework.
(5) Australian Digital Health Agency (2021). Workforce Strategy 2021–2026.
(6) Australasian Institute of Digital Health (2022). Australian Health Informatics Competency Framework.
(7) Australasian Institute of Digital Health (2022). Australian Digital Health Executive Competencies: Second Edition.
(8) Australian Medical Council (2021). Digital Health in Medicine Capability Framework.
(9) Royal Australian College of General Practitioners (2021). RACGP educational framework.
(10) Royal Australasian College of Medical Administrators (2011). Medical Leadership and Management Curriculum Framework.
(11) Royal Australasian College of Physicians (2013). Physician and Paediatrician Training Program Professional Qualities Curriculum.
(12) Victoria Health (2021). Digital Health Capability Framework for Allied Health Professionals.

## Appendix B. Postgraduate Healthcare Management Programs Analyzed

The 17 contemporary postgraduate health management programs offered domestically in Australia were analyzed, including publicly available information from:

(1) Charles Sturt University—Master of Health Management and Leadership: https://study.csu.edu.au/courses/medical-science/master-health-services-management (accessed on 2 September 2022).
(2) Curtin University—Master of Health Administration: https://handbook.curtin.edu.au/courses/course-pg-master-of-health-administration--mc-hladmnv1 (accessed on 2 September 2022).
(3) Deakin University—Master of Business Administration (Healthcare Management): https://www.deakin.edu.au/course/master-business-administration-healthcare-management (accessed on 2 September 2022).
(4) Deakin University—Master of Health and Human Services Management: https://www.deakin.edu.au/course/master-health-and-human-services-management (accessed on 2 September 2022).
(5) Flinders University—Master of Health Administration: https://www.flinders.edu.au/study/courses/postgraduate-health-administration (accessed on 2 September 2022).
(6) Flinders University—Master of Business Administration (Healthcare Management): https://www.flinders.edu.au/study/courses/postgraduate-business-administration-health-management (accessed on 2 September 2022).
(7) Griffith University—Master of Health Services Management: https://www.griffith.edu.au/study/degrees/master-of-health-services-management-5586 (accessed on 2 September 2022).
(8) Latrobe University—Master of Health Administration: https://www.latrobe.edu.au/courses/master-of-health-administration (accessed on 2 September 2022).
(9) Monash University—Master of Health Administration: https://online.monash.edu/online-courses/health-courses/online-master-health-administration (accessed on 2 September 2022).
(10) Murdoch University—Master of Health Care Management: https://www.murdoch.edu.au/course/Postgraduate/M1217 (accessed on 2 September 2022).
(11) Queensland University of Technology—Master of Health Management: https://online.qut.edu.au/online-courses/health/master-of-health-management/ (accessed on 2 September 2022).
(12) University of Adelaide—Master of Business Administration Health Management: https://online.adelaide.edu.au/campaign-lp-master-of-business-administration-health-management (accessed on 2 September 2022).
(13) University of New England—Master of Health Management: https://handbook.une.edu.au/courses/2022 (accessed on 2 September 2022).
(14) University of New South Wales—Master of Health Leadership and Management: https://www.unsw.edu.au/study/postgraduate/master-of-health-leadership-and-management (accessed on 2 September 2022).
(15) University of Tasmania—Master of Health Service Management: https://www.utas.edu.au/courses/bus/courses/c7o-master-of-health-service-management (accessed on 2 September 2022).
(16) University of Technology Sydney—Master of Health Services Management: https://studyonline.uts.edu.au/online-courses/master-health-services-management (accessed on 2 September 2022).
(17) Western Sydney University—Master of Health Science (Health Service Management): https://www.westernsydney.edu.au/future/study/courses/postgraduate/master-of-health-science-health-services-management (accessed on 2 September 2022).
